# Dietary fiber sources and non-starch polysaccharide-degrading enzymes modify mucin expression and the immune profile of the swine ileum

**DOI:** 10.1371/journal.pone.0207196

**Published:** 2018-11-08

**Authors:** Marta Ferrandis Vila, Michaela P. Trudeau, Yuan-Tai Hung, Zhikai Zeng, Pedro E. Urriola, Gerald C. Shurson, Milena Saqui-Salces

**Affiliations:** 1 Department of Animal Science, University of Minnesota, St. Paul, Minnesota, United States of America; 2 Veterinary Population Medicine, University of Minnesota, St. Paul, Minnesota, United States of America; University of Illinois, UNITED STATES

## Abstract

Due to their complex chemical and physical properties, the effects and mechanisms of action of natural sources of dietary fiber on the intestine are unclear. Pigs are commonly fed high-fiber diets to reduce production costs and non-starch polysaccharide (NSP)-degrading enzymes have been used to increase fiber digestibility. We evaluated the expression of *mucin 2* (*MUC2*), presence of goblet cells, and ileal immune profile of pigs housed individually for 28 days and fed either a low fiber diet based on corn-soybean meal (CSB, n = 9), or two high fiber diets formulated adding 40% corn distillers’ dried grains with solubles (DDGS, n = 9) or 30% wheat middlings (WM, n = 9) to CSB-based diet. Pigs were also fed those diets supplemented with a NSP enzymes mix (E) of xylanase, β-glucanase, mannanase, and galactosidase (n = 8, 10, and 9 for CSB+E, DDGS+E and WM+E, respectively). Feeding DDGS and WM diets increased ileal *MUC2* expression compared with CSB diet, and this effect was reversed by the addition of enzymes. There were no differences in abundance of goblet cells among treatments. In general, enzyme supplementation increased gene expression and concentrations of IL-1β, and reduced the concentrations of IL-4, IL-17A and IL-11. The effects of diet-induced cytokines on modulating intestinal *MUC2* were assessed *in vitro* by treating mouse and swine enteroids with 1 ng/ml of IL-4 and IL-1β. In accordance with previous studies, treatment with Il-4 induced *Muc2* and expansion of goblet cells in mouse enteroids. However, swine enteroids did not change *MUC2* expression or number of goblet cells when treated with IL-4 or IL-1β. Our results suggest that mucin and immune profile are regulated by diet in the swine intestine, but by mechanisms different to mouse, emphasizing the need for using appropriate models to study responses to dietary fiber in swine.

## Introduction

Dietary fiber is one of the most significant factors that affect gut physiology and health in humans [1] and livestock [2,3]. It is generally agreed that dietary fiber is needed to maintain normal intestinal function [4], promote gastrointestinal health, increase satiety, and improve animal welfare [5,6]. In humans, the recommended daily intake of dietary fiber is based on protective effects of fiber on the development of cardiovascular disease [1], but there are no recommended levels of dietary fiber intake defined for optimal gastrointestinal function for humans or domestic animals, including swine. Although whole grains and grain by-products are routinely fed to livestock, and are comprised of a complex mixture of different types and amounts carbohydrates, most studies in the literature have used purified sources of fiber in experimental diets. The specific intestinal responses to natural sources of fiber are not defined at the molecular level, and present a significant challenge because the effects observed can vary depending on the chemical and physical properties of the specific fiber sources being fed to animals [4].

In general, feeding diets containing relatively high concentrations of dietary fiber results in an increase in intestinal mucin production [7,8]. This mucus layer has an important protective function by acting as a barrier between the luminal contents and the absorptive system of the intestine to protect the epithelium from luminal insults and disease [8–10]. In a previous study, we showed that fiber sources with high insoluble fiber content commonly used in swine feeds (i.e. corn distiller’s dried grains with solubles—DDGS, soybean hulls, and wheat straw) increased *MUCIN 2* (*MUC2*) gene expression and the number of goblet cells in the swine intestine [8]. Other researchers have shown that certain cytokines, like IL-4 and IL-13, have the ability to modulate mucin secretion [11,12], suggesting that cytokines in the intestine may participate in the intestinal changes induced by fiber.

Non-starch polysaccharide (NSP)-degrading enzymes are added to diets for food producing animals to increase the energy obtained from diets with high fiber content. It has been reported that the addition of NSP-degrading enzymes improve animal growth performance, nutrient digestibility and responses to infectious diseases [13–15]. However, the mechanisms by which NSP-degrading enzymes exert these effects are unclear. In this study, we analyzed the effect of feeding high-fiber ingredients commonly used in swine diets (i.e. DDGS and wheat middlings—WM), compared with feeding a standard corn-soybean meal (CSB) based diet, on *mucin* expression and the intestinal immune profile. We also added a NSP-degrading enzyme cocktail to these diets to determine their effect on these parameters, and evaluated the potential role of fiber-induced cytokines on the regulation of mucin secretion *in vitro* using enteroids.

## Materials and methods

### Animals

All animal protocols were reviewed and approved by the University of Minnesota Institution Animal Care and Use Committee (IACUC). For the swine feeding trial (IACUC project #1604-33628A), fifty-four pigs (initial body weight = 25.33 ± 0.41 kg) that were offspring of Yorkshire × Landrace sows (TOPIGS USA, Des Moines, IA) sired by Duroc boars (Compart Boar Store, Nicollet, MN) were housed in individual pens (1.5 x 1.5 m) at the University of Minnesota Southern Research and Outreach Center (Waseca, MN) and assigned randomly to 1 of 6 dietary treatments to provide a total of 9 pigs (5 barrows and 4 gilts) per treatment. Five to ten-week-old C57BL/6J mice and adult pigs were used to harvest stem cells to generate enteroids. Animals were housed and handled under standard conditions at the University of Minnesota St. Paul Campus (IACUC projects 1604-33628A and 1606-33871A).

### Swine experiment

Pigs were fed one of six diets consisting of 1) control diet based on corn and soybean meal (CSB), 2) CSB containing 40% corn distillers dried grains with solubles (DDGS) 3) CSB containing 30% wheat middlings (WM), and the same diets supplemented with 100 mg/kg of an exogenous NSP-degrading enzyme cocktail (E, Archer Daniels Midland Company, ADM, Decatur, IL) (CSB+E, DDGS+E, WM+E; Table 1). The enzyme cocktail was composed of 1500 U/g xylanase, 1100 U/g β-glucanase, 110 U/g mannanase, and 35 U/g galactosidase. All diets were fed in mash form and contained titanium dioxide (0.5%) and supplemental phytase (1,000 FTU/kg, Quantum, AB Vista, Plantation, FL) that supplied an equivalent of 0.1% calcium and 0.12% digestible phosphorus to the diets. Diets were formulated to meet or exceed nutritional requirements for 25 kg pigs fed diets containing 3,300 kcal/kg of metabolizable energy [16].

**Table 1:** Diets formulation and calculated nutrient content.

Item	CSB	DDGS	WM
*Ingredient composition (%)*			
Yellow dent corn	72.00	42.02	46.66
Soybean meal	25.00	15.00	18.00
Corn distillers dried grains with solubles (DDGS)	-	40.00	-
Wheat middling	-	-	30.00
Soybean oil	-		2.23
Dicalcium phosphate	0.30	-	-
Limestone	1.36	1.66	1.56
Salt	0.25	0.25	0.25
L-Lys HCl 78%	0.27	0.35	0.38
DL-Met 98%	0.06	-	0.09
L-Thr 98%	0.05	-	0.12
L-Trp 99%	-	0.01	-
Phytase 10,000 FTU/g	0.01	0.01	0.01
Vitamin premix^1^	0.25	0.25	0.25
Mineral premix^2^	0.15	0.15	0.15
Titanium dioxide 40% Ti	0.30	0.30	0.30
Total	100	100	100
*Calculated nutrient composition*			
ME (Kcal/kg)	3,285	3,295	3,285
NE (Kcal/kg)	2,446	2,373	2,425
CP (%)	18.18	22.76	17.56
Ether extract (%)	2.89	5.10	5.14
NDF (%)	8.61	17.30	17.22
ADF (%)	3.39	6.15	5.78
Total Ca (%)	0.66	0.66	0.66
Total P	0.42	0.59	0.56
*Standardized total tract digestible P*	0.31	0.45	0.40
*Standardized ileal digestible AA (%)*			
Lys	1.00	1.00	1.00
Met + Cys	0.57	0.71	0.57
Thr	0.60	0.67	0.60
Trp	0.18	0.18	0.18
Val	0.71	0.86	0.65

^1,2^ The premix provided the following per kilogram of complete diet: vitamin A, 12,000 IU; vitamin D3, 2,500 IU; vitamin E, 30 IU; vitamin K3, 3 mg; vitamin B12, 0.012 mg; riboflavin, 4 mg; niacin, 40 mg; pantothenic acid, 15 mg; choline chloride, 400 mg; folic acid, 0.7 mg; thiamin, 1.5 mg; pyridoxine, 3 mg; biotin, 0.1 mg; Zn, 105 mg; Mn, 22 mg; Fe, 84 mg; Cu, 10 mg; I, 0.50 mg; Se, 0.35 mg.

Pigs were provided *ad libitum* access to experimental diets and water for 28 days. Before euthanasia, pigs were fasted for 8 h followed by providing *ad libitum* access to feed for 12 h [17]. Ileal tissue samples were collected at a location that was 15 cm proximal to the ileocecal valve, and were either fixed in 4% formalin, processed and paraffin embedded, or snap frozen in liquid nitrogen and stored at -80°C until further analysis.

### Goblet cell quantitation

Ileal tissue samples fixed in formalin were processed and embedded in paraffin for histology. Four-micron sections of ileal tissues and treated enteroids were cut and stained with periodic acid Schiff–Alcian blue (PAS-AB, Newcomer Supply, Middleton, WI). In ileal tissues, presence of goblet cells were estimated as the percentage of the mucosal area that was positive for PAS-AB as previously described [8]. Total numbers of cells and PAS positive goblet cells were counted in all enteroids observed in 5 sections collected each 20 μm apart for each treatment under light microscopy at 60X magnification.

### Gene expression

Total RNA from the ileal tissue samples was isolated using the RNeasy Plus Universal Mini Kit (Qiagen, Valencia, CA) following the manufacturer’s instructions. Total RNA from the enteroids was purified using the RNeasy Plus Universal Micro Kit (Qiagen, Valencia, CA) after collecting and pelleting enteroids by centrifugation, and lysis using TRIzol reagent followed chloroform and isopropanol RNA extraction. The RNA was quantified using a NanoDrop 2000 instrument (Thermo Scientific, Wilmington, DE), and 500 ng of RNA were reverse transcribed using the High Capacity cDNA reverse Transcription Kit (Applied Biosystems, Foster City, CA).

In swine ileum tissues, the gene expression of *interferon gamma* (*IFNγ*), *tumor necrosis factor alpha* (*TNFα*), *interleukin* (*IL*) *1β* (*IL-1β*), *IL-4*, *IL-6*, *IL-8*, *IL-10*, *IL-11*, *IL-12p40*, *IL-17A*, *IL-23A*, *IL-25*, *mucin 2* (*MUC2*), *glyceraldehyde 3-phosphate dehydrogenase* (*GAPDH*), and *hypoxanthine-guanine phosphoribosyl transferase* (*HPRT*) were determined. In enteroids samples, the gene expression of *Mucin 2* (*Muc2*), *glyceraldehyde 3-phosphate dehydrogenase* (*Gapdh*) and *hypoxanthine-guanine phosphoribosyl transferase* (*Hprt*) were determined. Quantitative PCR was performed using Power SYBR Green PCR Master Mix (Applied Biosystems, Foster City, CA) in a Quantum Studio 3 system (Applied Biosystems, Foster City, CA). The PCR conditions used were: initial activation at 95°C for 10 min, followed by 40 cycles of 95°C for 15 sec denaturation, and annealing at 60°C for 60 secs. The primer sequences used are shown in Table 2. Relative gene expression was calculated using the primer efficiency values as described by Pfaffl [18], with Ct values > 38 considered as non-detectable. Housekeeping genes *Gapdh* and *Hprt* were used as reference genes for both swine and mouse experiments.

**Table 2:** Sequences of primers used in this study for cytokine profiling.

Gene	Forward Sequence	Reverse Sequence	Reference
Swine			
*IFNγ*	GCTTTTCAGCTTTGCGTGACT	TCACTCTCCTCTTTCCAATTCTTC	This study
*TNFα*	AGCACTGAGAGCATGATCCG	GACATTGGCTACAACGTGGG	This study
*IL-1β*	CCAATTCAGGGACCCTACC	CATGGCTGCTTCAGAAACCT	[19]
*IL-4*	CCAACCCTGGTCTGCTTACTG	TTGTAAGGTGATGTCGCACTTGT	[20]
*IL-6*	TGAACTCCCTCTCCACAAGC	GGCAGTAGCCATCACCAGA	[19]
*IL-8*	AAGCTTGTCAATGGAAAAGAG	CTGTTGTTGTTGCTTCTCAG	[21]
*IL-10*	CACTGCTCTATTGCCTGATCTTCC	AAACTCTTCACTGGGCCGAAG	[22]
*IL-11*	CAAATTCCCAGCTGACGGAGA	GTAGGAAAACAGGTCTGCTCG	
*IL-12p40*	GAGGGTGAGTGAGTGCCTTG	ACTCCGCCTAGGTTCGACTT	[19]
*IL-17A*	ATCCTCGTCCCTGTCACTGC	ACATGCTGAGGGAAGTTCTTGTC	[23]
*IL-23A*	CCAAGAGAAGAGGGAGATGATGA	TGCAAGCAGGACTGACTGTTGT	[24]
*IL-25*	GAACCCACACCTTCCATTTG	ATCTCCAGAGGAGGCATGAG	[25]
*MUC2*	GGCTGCTCATTGAGAGGAGT	ATGTTCCCGAACTCCAAGG	[8]
*GAPDH*	ATCCTGGGCTACACTGAGGAC	AAGTGGTCGTTGAGGGCAATG	[26]
*HPRT*	GGACTTGAATCATGTTTGTG	CAGATGTTTCCAAACTCAAC	[27]
Mouse			
*Hprt*	AGGACCTCTCGAAGTGTTGGATAC	AACTTGCGCTCATCTTAGGCTTTG	[28]
*Gapdh*	TCAAGAAGGTGGTGAAGCAGG	TATTATGGGGGTCTGGGATGG	[29]
*Muc2*	AGAACGATGCCTACACCAAG	CATTGAAGTCCCCGCAGAG	[29]

### Cytokine tissue levels

Tissue protein was extracted by homogenization of ileal samples in lysis buffer containing deoxycholic acid (12.7 mM; Sigma Aldrich), Igepal CA-630 (1%; Sigma Aldrich), Tris-HCl (50mM; Sigma Aldrich), NaCl (150mM; Sigma Aldrich), and protease inhibitor cocktail (1x, Halt protease inhibitor Cocktail, Thermo Fischer Scientific, Rockford, IL) adjusted to pH 7.4. Homogenized samples were centrifuged at 12,000 x *g* for 15 min at 4°C. Total protein was quantified using a NanoDrop 2000 instrument (Thermo Scientific, Wilmington, DE). Samples of each group were then adjusted to the same protein concentration and pooled (four or five) for cytokine analysis. A Multiplex Map Kit (Porcine cytokine/chemokine Magnetic Bead Panel, Merck Millipore, Darmstadt, Germany) was used to quantify IL-1β and IL-4. Cytokine concentrations of IL-17A were measured using a RayBio Porcine IL-17 ELISA kit (Raybiotech Inc., Monterouge, France). Concentration of IL-11 was measured using the Nori Porcine IL-11 ELISA kit (Genorise Scientific Inc., Philadelphia, USA) and concentrations of IL-25 were determined using a Nori Porcine IL-25/IL-17E ELISA kit (Genorise Scientific Inc., Philadelphia, USA). All ELISA kits were used following manufacturer’s instructions.

### Crypt isolation and enteroid culture

Enteroids were cultured from mouse isolated crypts obtained following the protocols described by Mahe et al. [30], with culture media formulated with advanced DMEM/F12 (Gibco, Thermo Fisher Scientific, Waltham, MA) supplemented with 2mM GlutaMax (Gibco), 10 mM HEPES (Gibco), 100 U/mL penicillin/100 μg/mL streptomycin (Gibco), 1× N2 (Gibco) and 1× B27 (Gibco) supplements, 100 ng/mL Noggin (Gibco), 50 ng/mL epithelial growth factor (R&D Systems, Minneapolis, MN), 5% R-spondin, 5% Wnt-3a conditioned media obtained from transformed Hek293 cells expressing R-spondin (a kind gift from Dr. Jason Spence, University of Michigan), and Wnt-3a L-cells (ATTCC CRL-2647). Enteroids were cultured in humidified incubators at 37°C and 5% CO_2_.

Swine enteroids were cultured from isolated crypts from adult swine ileum. Briefly, a piece of ileum of 2 to 3 cm was obtained at the time of euthanasia, washed three times in phosphate saline buffer with 1% antibiotic/antimycotic (Gibco), followed by dissociation in Hank’s balanced salt solution (Ca and Mg free, Gibco) with 30 mM ethylenediaminetretraacetic acid (Sigma Aldrich), 1mM dithiothreitol (Sigma Aldrich), and 1% penicillin/streptomycin (Gibco) at 37°C with agitation for 10–15 min, or until isolated crypts were visible. The crypts were centrifuged at 400 x *g* for 5 min and washed once with DMEM/F12. After the last centrifugation, the pelleted crypts were re-suspended in a small volume of DMEM/F12, mixed with Matrigel (Corning) in 1:3 ratio, and plated as beads in 24-well plates (Corning). Once Matrigel hardened, beads were covered with 500 μl of Intesticult organoid growth medium (Stemcell Technologies, Vancouver, CAN) and cultured in humidified incubator at 39°C and 5% CO_2_.

### Enteroid treatment with interleukins

Recombinant mouse Il-1β, Il-4, Il-11, and Il-17A were purchased from PeproTech (Rocky Hill, NJ) and swine recombinant IL-4 and IL-1β were purchased from ImmunoChemisty Technologies (Bloomington, MN). All interleukins were diluted in sterile water containing 0.1% bovine serum albumin (Sigma Aldrich) as a carrier. Mouse and swine enteroids were plated with an average density of 15 enteroids/well. Mouse enteroids were maintained in differentiation media by removing Wnt3a from media for 3 days before applying the treatments. Enteroids were stimulated with 500 μl of media containing 1 ng/mL of the corresponding interleukin or vehicle (control). Experiments were repeated three times with replicate treatments in each experiment, and samples were collected after 24, 48 and 72 h of treatment. For the 48 and 72 h treatments, medium was replenished every 24 h. Treated enteroids were collected in Trizol for RNA extraction, or fixed in 4% buffered paraformaldehyde for 2 h at room temperature, and then embedded in Histogel (Thermo Scientific), processed and paraffin embedded.

### Statistical analysis

All data were tested for normality using D’Agostino and Pearson tests. For the enteroids trial, data analyzed were the result of three independent experiments. Data were analyzed using ANOVA followed by Tukey’s or Dunn’s test using Prism 7.04 (GraphPad Software, Inc., La Jolla, CA, USA) software.

## Results and discussion

### High-fiber diets increased the expression of ileal mucin without affecting the proportion of goblet cells

**Fig 1:**
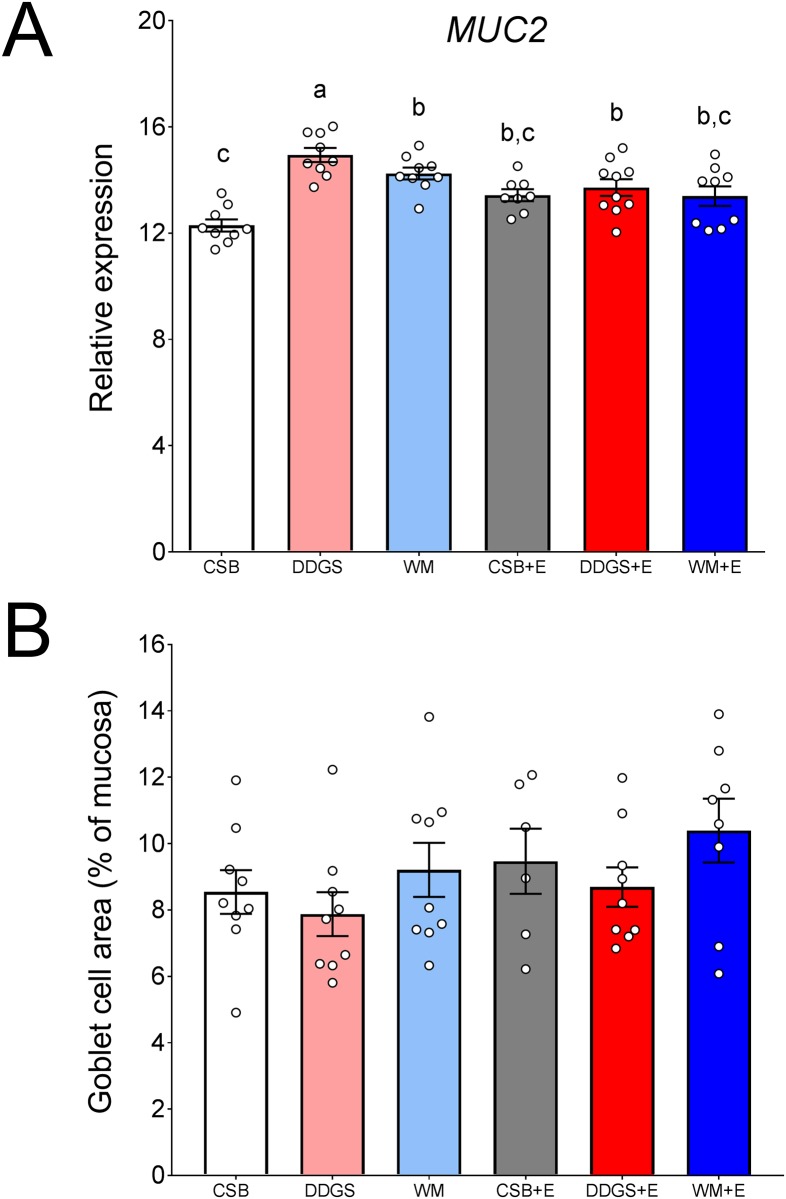
Expression of *MUC2* (A) and proportion of goblet cells (B) in the ileum of pigs fed high-fiber diets, with or without carbohydrase enzyme cocktail supplementation, for 28 days. Data are presented as mean ± standard error of the mean. Treatment groups with different superscripts are different (*P* > 0.05).

Expression of *MUC2* was increased (*P* ≤ 0.0001) by feeding the high-fiber diets (DDGS and WM) compared with feeding the CSB diet, and was greater (*P* < 0.05) in pigs consuming DDGS than WM (Fig 1A). Addition of the enzymatic cocktail did not change the *MUC2* expression when the CBS+E or WM+E diets were fed, compared with their respective non-enzyme supplemented diets. Although the enzyme cocktail reduced *MUC2* expression in pigs fed the DDGS diet (*P* = 0.029 for DDGS *vs*. DDGS+E), *MUC2* expression in the ileum of pigs fed DDGS+E was still greater than those fed CSB (*P* = 0.007). To determine if changes in *MUC2* expression correspond to increased presence of goblet cells in the ileum, tissue sections were stained for PAS and the area of the mucosa occupied by goblet cells was quantified. We found no differences in the amount of goblet cells among dietary treatments (Fig 1B).

The reduction of the presence of mucus and *Muc2* expression in the intestine of animals from feeding different high-fiber diets containing NSP-degrading enzyme cocktails has been previously reported in turkeys and broilers [31,32]. However, we are unaware of any studies that have evaluated the presence of goblet cells when feeding high fiber diets with NSP-degrading enzymes. One study showed that supplementing broiler diets with increasing dietary concentrations of β-mannanase resulted in a reduction of the number of goblet cells in the swine small intestine [13]. The results of our study partially support our previous finding of increased *MUC2* expression in the ileum in pigs fed high-fiber diets [8]. In that study however, we also observed increased goblet cell area in the ileum of pigs fed corn-soybean meal diets were supplemented with 23% wheat straw and 55% DDGS. There are many possible reasons for this discrepancy, among them is that the diets in the two studies were different in fiber inclusion level and composition. Also, adaptation to the diet may result in stronger effects at earlier time points (14 days in our previous study). Another important difference between both studies is feed intake. In our previous study, pigs were fed 2.5% of their initial body weight two times a day, while the present study allowed *ad libitum* access to feed. The impact of feed intake and adaptation to diets on intestinal mucins and goblet cells requires further investigation.

Other researchers have reported that feeding a diet containing 30% DDGS, with or without supplemental xylanases, did not change the pig ileal expression of *MUC4* or *MUC20* [14]. Studies in rats have suggested that, at least for soluble fiber sources, an increase in *Muc2* expression may not be always associated with increased number of goblet cells, and these differences may be due to the specific chemical characteristics of the fiber source fed, the amount of fiber in the diets, and days of adaptation to high fiber diets before tissue collection [33,34]. It is unknown whether the disparate observations of increased mucin expressions and goblet cell numbers depend only of fiber characteristics or are species-specific responses to fiber.

### Fiber sources and enzymatic supplementation affect the ileal immune profile

To evaluate the effects of feeding fiber from DDGS and WM, along with NSP-degrading enzyme on the immune response of the pig intestine, we examined the ileal gene expression of twelve cytokines to identify possible changes in pro- and anti-inflammatory, as well as regulatory cytokines. No differences were observed in the expression of *IFNγ*, *TNFα*, *IL-2*, *IL-6*, *IL-8*, *IL-10*, *IL-12p40*, and *IL-23* induced by feeding high-fiber diets or enzyme supplementation (Table 3). Feeding high fiber diets did not result in changes in *IL-1β* expression. However, the addition of this NSP-degrading enzyme increased *IL-1β* expression in the high-fiber diets (*P* < 0.001 for DDGS+E and WM+E compared with DDGS and WM, respectively). Pigs fed the WM diet had greater concentrations of *IL-4* compared with those fed the DDGS (*P* = 0.016) and DDGS+E diets (*P* = 0.025). Addition of the enzyme cocktail, however, reversed the effect so that *IL-4* expression in pigs fed WM+E was less than in pigs fed WM (*P* = 0.006), but not different than that of pigs fed other diets. The expression of *IL-11* was less in pigs fed the WM diet compared with those fed CSB (*P* = 0.0003) and DDGS (*P* = 0.003) diets. The addition of enzyme cocktail drastically reduced *IL-11* expression (*P* < 0.0001) in pigs fed the DDGS+E and WM+E diets compared with feeding the same diets without enzyme supplementation. Compared to pigs fed diets without enzymes, those fed enzyme supplemented diets showed no differences on *IL-17A* expression. However, pigs fed WM+E had reduced expression of *IL-17A* than pigs fed CSB (*P* = 0.026) and DDGS (*P* = 0.012). Expression of *IL-25* was greater in pigs fed WM compared with those fed CSB (*P* = 0.006). However, there were no differences in *IL-25* expression among pigs fed diets with this enzyme cocktail, but pigs fed DDGS+E showed greater expression when compared with those fed CSB (*P* = 0.035).

**Table 3:** Relative gene expression of cytokines in the ileum of pigs fed high-fiber diets with or without enzyme supplementation for 28 days.

	Diet						
Cytokine	CSB	DDGS	WM	CSB+E	DDGS+E	WM+E	*P*-value
*IFNγ*	0.069±0.008	0.065±0.009	0.069±0.01	0.069±0.008	0.068±0.007	0.071±0.01	0.89
*TNFα*	97±29.7	107.8±47.9	95.6±15.7	86.2±12.9	82.8±27.3	101.6±52.1	0.64
*IL-1β*	0.059±0.006^c^	0.059±0.01^c^	0.061±0.007^c^	0.071±0.02^b,c^	0.086±0.01^a,b^	0.091±0.01^a^	**<0.001**
*IL-2*	1.95±0.09	1.99±0.14	1.92±0.04	1.94±0.08	1.87±0.05	1.91±0.11	0.13
*IL-4*	0.035±0.006^a,b^	0.032±0.004^b^	0.043±0.01^a^	0.033±0.004^a,b^	0.032±0.007^b^	0.031±0.003^b^	**0.006**
*IL-6*	0.028±0.004	0.027±0.007	0.024±0.003	0.025±0.004	0.023±0.003	0.025±0.003	0.22
*IL-8*	12.3±0.9	12.5±1.2	11.7±1.1	11.7±1.3	11.6±1.1	11.4±1.5	0.34
*IL-10*	1.98±0.08	1.99±0.1	1.97±0.04	1.96±0.07	1.92±0.04	1.94±0.08	0.38
*IL-11*	459.5±107^a^	435.8±77^a^	297.9±81^b^	422.4±79^a^	196.7±40^c^	140.4±21^c^	**<0.001**
*IL-12p40*	0.0051±0.001	0.0052±0.001	0.0054±0.001	0.0052±0.000	0.0062±0.001	0.0059±0.000	0.08
*IL-17A*	675±110^a^	692.4±159^a^	590.3±107^a,b^	548.3±82^a,b^	575.6±165^a,b^	486.8±82^b^	**0.008**
*IL-23A*	479.7±99	502.3±155	513.3±101	521.4±168	530.5±133	614.9±230	0.54
*IL-25*	0.13±0.02^b^	0.14±0.01^a,b^	0.17±0.02^a^	0.15±0.01^a,b^	0.16±0.02^a^	0.15±0.02^a,b^	**0.007**

Relative expression data are mean ± standard deviation. Different letter superscripts within each row differ (*P* < 0.05).

To validate if gene expression changes observed resulted in changes in protein content in the tissue, we analyzed ileum tissue samples for the concentration of the interleukins of interest (Table 4). Overall, IL-1β levels were greater in the pigs fed the enzyme supplemented diets than the non-supplemented diets (*P* < 0.02). Pigs fed WM+E had the greatest IL-1β concentrations of all dietary treatments (WM+E vs. CSB+E, *P* = 0.012; WM+E vs. DDGS+E, *P* = 0.019; WM+E *vs*. all non-supplemented diets, *P* < 0.001). The IL-4 levels were greater in the WM fed group compared with all other diets (*P* < 0.001), and this effect was reversed by enzyme supplementation (WM vs. WM+E, *P* < 0.0001). A similar effect, but of less magnitude, was observed for feeding CSB compared with CSB+E diets (*P* = 0.023). The concentration of IL-11 was reduced in all diets with enzyme supplementation (*P* < 0.005), and was independent of the dietary fiber content or source. The IL-17A concentration was decreased by enzymatic treatment in high-fiber diets (DDGS+E vs. DDGS, *P* = 0.010; WM+E vs. WM, *P* = 0.025). However, no changes in tissue concentrations of IL-25 were observed.

**Table 4:** Concentration (pg/mg total protein) of cytokines in ileal tissue of pigs fed high fiber diets with or without enzyme supplementation for 28 days.

Diet							
	CSB	DDGS	WM	CSB+E	DDGS+E	WM+E	*P*-value
IL-1β	1.388±0.16^c^	1.565±0.22^c^	1.502±0.08^c^	2.143±0.16^b^	2.232±0.32^b^	2.887±0.23^a^	**0.002**
IL-4	0.002±0.01^b^	0.001±0.01^b,c^	0.004±0.01^a^	0.0008±0.01^c^	0.001±0.01^b,c^	0.001±0.01^b,c^	**0.042**
IL-11	0.018±0.01^a^	0.017±0.01^a^	0.014±0.02^a^	0.008±0.02^b^	0.005±0.01^b^	0.005±0.01^b^	**0.001**
IL-17A	0.548±0.16^a^	0.646±0.13^a^	0.652±0.03^a^	0.432±0.03^a,b^	0.312±0.02^b^	0.385±0.07^b^	**0.039**
IL-25	0.440±0.09	0.377±0.06	0.304±0.08	0.369±0.06	0.368±0.13	0.387±0.004	0.710

Values presented as mean ± standard deviation. Different letter superscripts within each row differ (*P* < 0.05).

In general, the changes in gene expression levels were predictive of changes in protein in the tissue, which suggests that gene expression analysis can be used as a proxy for cytokine concentrations in the swine ileum. We found that the concentration of the cytokine IL-1β was greater in pigs fed diets containing NSP-degrading enzymes regardless the fiber type, but there was an opposite effect for IL-11 and IL-17A. A previous study by Weber et al. [35] reported an increase in ileal gene expression of *IL-6*, *IL-1β* and *IL-10* observed in piglets fed a 7.5% DDGS diet for seven days. We did not find changes in *IL-6* or *IL-10*, and we only observed changes in *IL-1β* when enzymes were added. Whether adaptation to diet can affect ileal gene expressions of cytokines deserved further investigation. Our results for IL-1β are in disagreement with a previous report showing that NSP degrading enzymes did not change expression of IL-1β or *MUC2* [36]. However, induction of pro-inflammatory cytokines such as IL-1β, TNFα and IL-6 has been reported after murine macrophage stimulation with semi-purified or purified NSP’s such as β-glucans [37,38], arabinogalactans [39], and α-glucans [40]. The suggested mechanism for these responses is that NSP’s bind to pattern recognition receptors (PRRs), which have high specificity for clustered glycans [41,42] and alters the expression of Toll-like receptor-induced cytokines [43,44]. Although those immunomodulatory effects have been determined by exposing isolated immune cells directly to purified NSPs, our study evaluated the intestinal immune profile resulting of exposure to complex fiber sources and NSP degrading enzymes. Therefore, a direct comparison of these contrasting results may be misleading. Our results overall show similar responses and suggest that there is a direct role of dietary fiber on the modulation of the intestinal immune profile.

It is commonly accepted, that the effects of fiber on the intestinal immune response depends not only on the fiber characteristics (i.e. solubility, fermentability and viscosity), but also on presence of proteins and lipids associated with high-fiber feed ingredients [2]. In addition, some fiber sources contain immune-modulatory compounds, such as yeast cell walls in DDGS, that are present as a result of using yeast to convert starch to ethanol during the fermentation process [45]. Various NSP’s from plants, algae, and fungi have immunological effects by stimulating different immune cells [46,47]. Furthermore, the addition of NSP degrading enzymes to high-fiber diets has been shown to alter intestinal epithelial integrity, immune related proteins [48], and reduce susceptibility to pathogenic bacteria infection [49]. Some of the suggested mechanisms for these responses to the NSP degrading enzyme products include, changes of the intestinal microbiota and production of short chain fatty acids [50]. However, because most of the commercially available NSP degrading enzymes are produced from bacteria, yeast or fungi, and are generally impure [51], it is possible that some of these chemical components may also affect the intestinal immune response.

### Effect of different cytokines on mucin expression

In the literature, changes in intestinal mucin expression have been associated to multiple factors including dietary fiber in general, insoluble and non-fermentable fiber, NSP content, protein in the diet, specific bacteria species, and inflammatory signals. To assess if the cytokines which concentrations were changed by the experimental diets participate in regulation of mucin expression levels, we exposed enteroids to these cytokines. We exposed mouse enteroids to Il-1β, Il-4, Il-11 and Il-17A, and swine ileal enteroids to IL-1β and IL-4.

**Fig 2:**
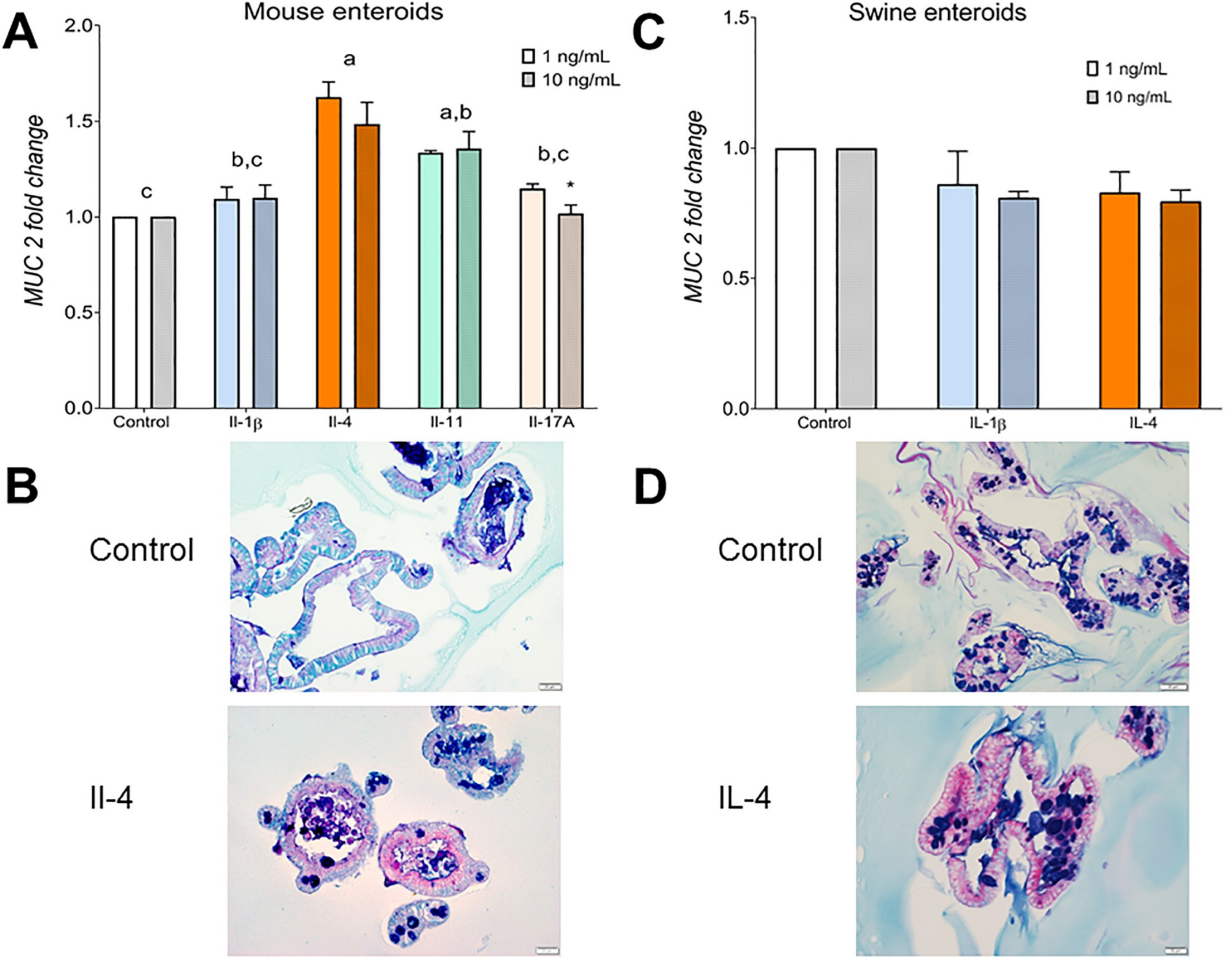
*Mucin 2* gene expression (A) and presence of goblet cells shown by staining with periodic acid-Schiff with Alcian blue (B) of mouse and swine enteroids (C and D) treated with different interleukins at 1 and 10 ng/mL concentration for 24 h. N = 3 independent experiments. Data are presented as mean ± standard error of the mean, normalized to the respective experimental control. Dietary treatment groups with different superscripts are different (*P* > 0.05). Asterisk (*) indicates different to Il-4 when treated with 10 ng/mL of Il-17A.

In the mouse enteroids, Il-4 induced the highest expression of *Muc2* compared to control (*P* = 0.002), Il-1β (*P* = 0.025, Fig 2A) and Il-17A (*P* = 0.044). Interleukin-eleven also caused an increase in *Muc2* expression compared with the control (*P* = 0.022), but it was not different compared with Il-1β or Il-4, and Il-17A was unaffected (*P* = 0.27). The effects of interleukins on *Muc 2* expression were observed during 24 h and 48 h with a trend to decrease by 72 h (S1 Fig) and were not affected by treatment dose (dose effect *P* = 0.22, interleukin effect *P* < 0.001, interaction *P* = 0.518). After 72 h of treatment, the proportion of goblet cells in the enteroids was increased by Il-4 (7.0 ± 0.05% goblet cells/total cell number) compared with the control (3.9 ± 0.03%, *P* = 0.013, Fig 2B) and Il-1β treatments (3.0 ± 0.02%, P = 0.001). Treatment with Il-11 (5.1 ± 0.03%) and Il-17A (6.0 ± 0.03%) did not change the number of goblet cells compared with the control (*P* = 0.23 and *P* = 0.075 respectively). Previous studies have linked increased concentrations of IL-4 and other cytokines (IL-13, TNFα) to augmented mucin secretion in the intestinal lumen [11,12] and the airway epithelia [52] in human and mouse models. With respect of Il-4 and Il-1β, our results were in agreement with those reported in previous studies [11,52]. We are not aware of any other studies that have reported the effects of Il-11 on mucin expression.

When swine enteroids were treated with IL-1β and IL-4 (Fig 2C), we found no differences on the gene expression of *MUC2* or in the number of goblet cells treated with 1 ng/mL interleukin concentration or 10 ng/mL (Fig 2B and 2D). The culture of swine enteroids, although challenging, has been previously reported in studies showing the presence of all cells of the intestine and their response to bacterial lipopolysaccharide stimulation [27,53,54]. The swine enteroids grown in our laboratory showed similar characteristics to those reported in previous studies, although we have defined that culturing at 39°C (the body temperature of pigs) favors the expression of differentiation markers, as has been previously suggested for adipocytes [55]. There is limited information in the literature about the regulation of mucin in swine. One study involved challenging pigs with laminarin, which resulted in increased *MUC2* ileal expression but no changes in INF-γ, IL-1α, IL-6, IL-8, IL-10 or TNF-α [56]. In a different study, providing epidermal growth factor (EGF) enterally to piglets resulted in increased gene expression of *IL-4*, *IL-13* and *MUC2* in the jejunum [57], suggesting that EGF and/or IL-13 may participate in the *MUC2* stimulation. Our finding that the effect of interleukins on the expression of *MUC2* in swine enteroids does not agree with the observations in mouse enteroids, may be explained by the known difference in function of IL-4 receptors between species [58,59], and reinforces the necessity of developing and validating *in vitro* models for the specific species and mechanisms of study.

## Conclusions

The results of this study showed that pigs fed high-fiber diets had increased ileal expression of *MUCIN 2*, without changes on the number of goblet cells. The addition of NSP-degrading enzymes modulates the local immune profile of the ileum, favoring a pro-inflammatory response. In contrast to the mouse, swine IL-4 does not promote *MUCIN 2* expression or goblet cells expansion, which suggests that other mechanisms may be responsible for mediated the effect of dietary fiber in swine.

## Supporting information

S1 Fig Gene expression of *Mucin 2* (*Muc 2*) of mouse enteroids treated with different doses of Il-1β, Il-4, Il-11 and Il-17A for 24, 48 and 72 h. Data presented are mean of two technical replicates.
(TIF)
