# Supplementary figures and images for: Dietary fiber sources and non-starch polysaccharide-degrading enzymes modify mucin expression and the immune profile of the swine ileum
Source: PLoS One. 2018 Nov 8;13(11):e0207196. doi: 10.1371/journal.pone.0207196 (PMC6224153; doi:10.1371/journal.pone.0207196)

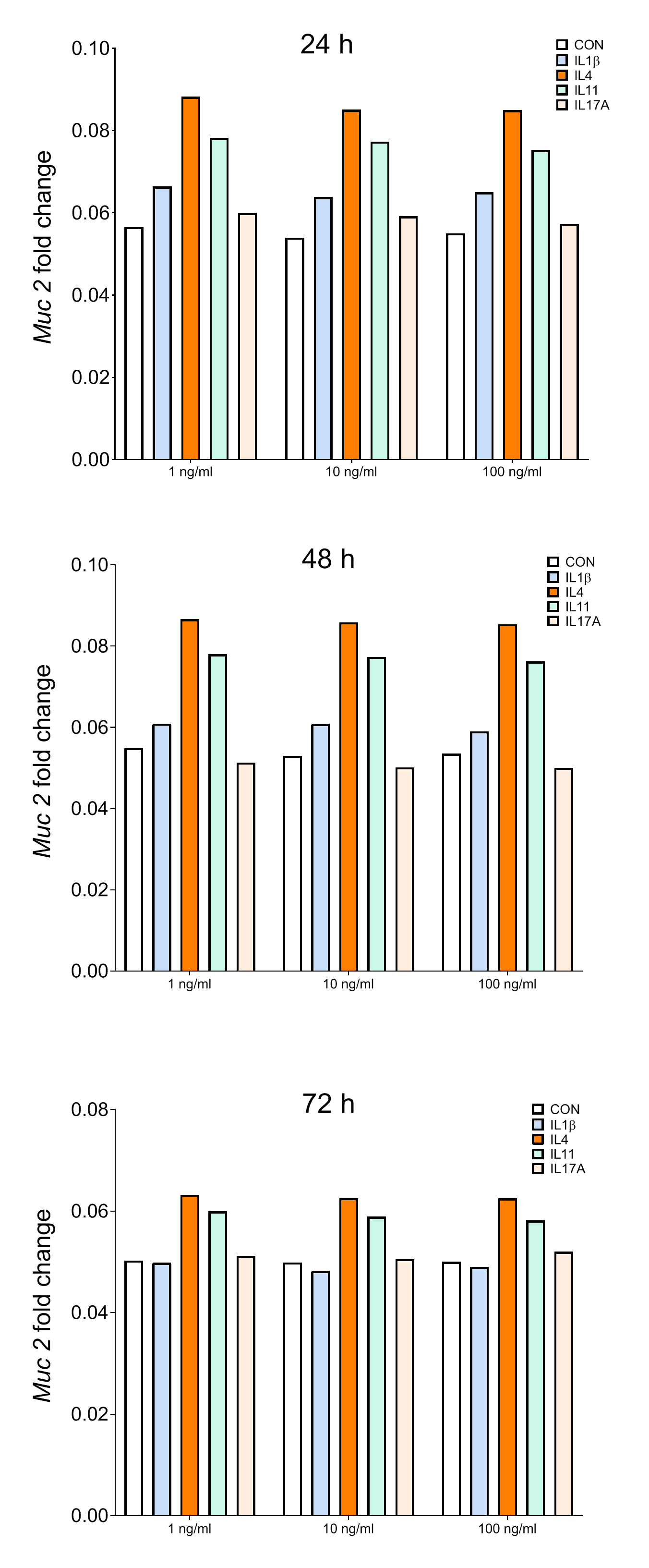

Supplement: S1 Fig — Data presented are mean of two technical replicates. (TIF) [file pone.0207196.s001.tif]
